# Progressive Posterior Lenticonus in a Patient with Alport Syndrome

**DOI:** 10.4103/0974-9233.71591

**Published:** 2010

**Authors:** Ammar M. Al-Mahmood, Samar A. Al-Swailem, Abdulrahman Al-Khalaf, Ghada Y. Al-Binali

**Affiliations:** Division of Anterior Segment, King Khaled Eye Specialist Hospital, Riyadh, Kingdom of Saudi Arabia; 1 Department of Ophthalmology, Bin Rushd Specialized Center, Riyadh, Kingdom of Saudi Arabia; 2 Department of Ophthalmology, Bahrain Defense Force Hospital, Kingdom of Bahrain

**Keywords:** Alport Syndrome, Anterior Lenticonus, Posterior Lenticonus, X-linked Disease

## Abstract

We report a rare case of Alport syndrome with progressive posterior lenticonus. A 24-year-old male presented to our tertiary eye care center with history of poor vision. At initial presentation, the patient had bilateral anterior lenticonus, posterior subcapsular cataract, and renal failure. The patient was diagnosed with Alport syndrome based on a positive family history of the disease and clinical findings. Further examination revealed progressive posterior lenticonus that was not present initially. The presence of such finding is important because it influences the surgical approach to avoid complications during cataract surgery.

## INTRODUCTION

Alport syndrome is a rare clinical entity characterized by the familial occurrence of hemorrhagic nephritis and sensorineural deafness (Alport 1927). Alport syndrome has a prevalence of 1/5000, with 85% of affected individuals having the X-linked form, where the affected males develop renal failure and usually have high-tone sensorineural deafness by the age of 20. The typical ocular signs are dot-and-fleck retinopathy, which occurs in 85% of the affected adult males, anterior lenticonus, which occurs in about 25%, and rare posterior polymorphous corneal dystrophy.^1^ Anterior lenticonus is considered a characteristic sign of Alport syndrome.^2^ Posterior lenticonus has been described as a rare manifestation in Alport syndrome^2^–^4^ and a review of the literature revealed a relative paucity of reports. We present here a case of Alport syndrome with anterior lenticonus and progressive posterior lenticonus.

## CASE REPORT

A 24-year-old male presented to our tertiary eye care hospital complaining of cloudy vision bilaterally since childhood, with the right eye affected more than the left. The patient had long-standing episodes of hematuria and was on renal dialysis for chronic renal failure at the time of initial presentation. In addition, the patient had reported difficulty in hearing.

**Figure 1:**
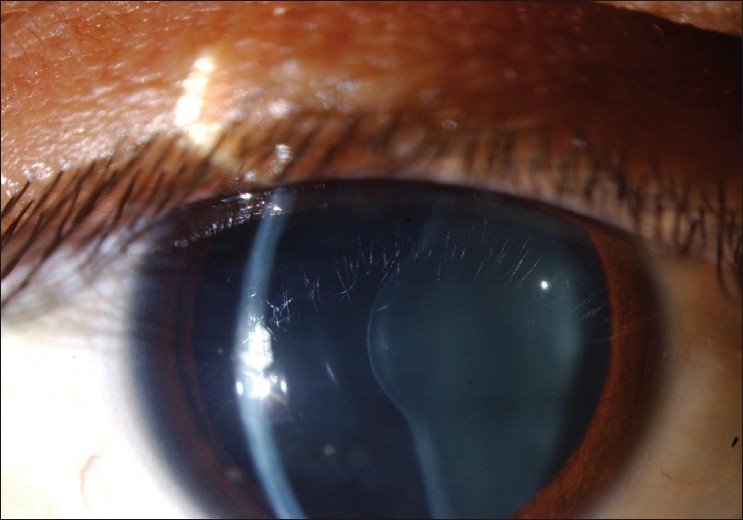
Anterior lenticonus in the right eye in a patient with Alport syndrome

**Figure 2:**
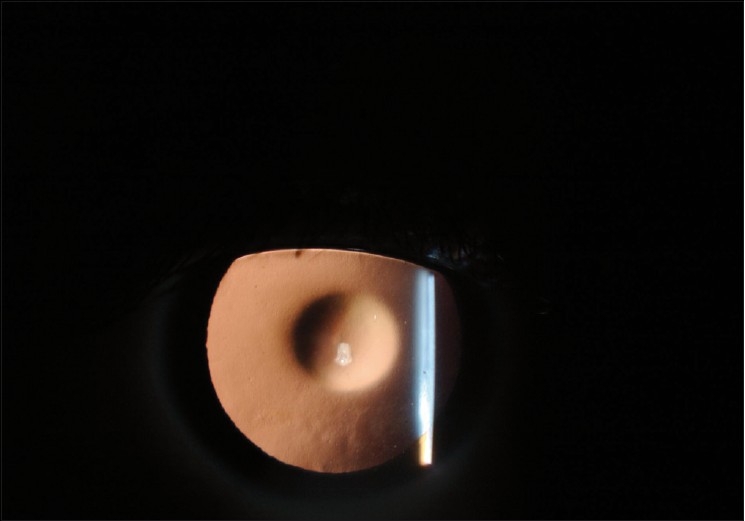
Oil droplet sign with retro-illumination in a patient with Alport syndrome

**Figure 3:**
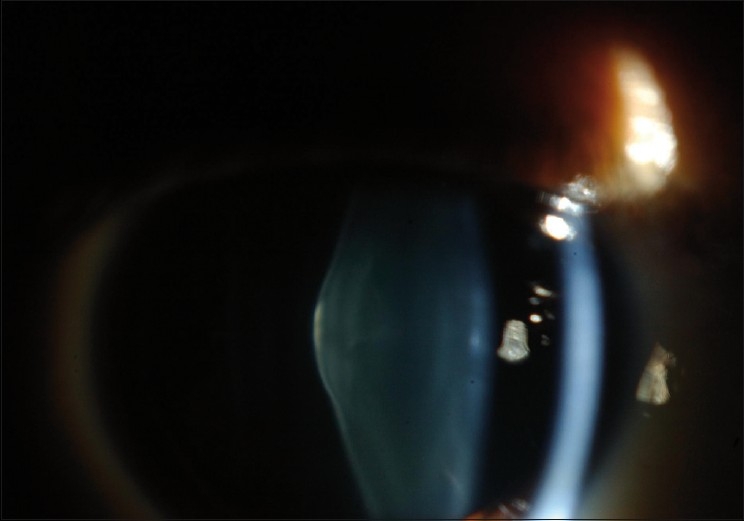
Posterior lenticonus in the right eye in a patient with Alport syndrome

Ocular examination revealed best corrected visual acuity of 20/80^+1^ in the right eye and 20/60^+1^ in the left eye. The patient’s manifest refraction was –2.50 –0.25 × 80 in the right eye and –2.50 –2.50 × 95 in the left eye. Intraocular pressure was normal bilaterally. Slit lamp examination of the right eye was within normal limits except for advanced anterior lenticonus, and fleck retinopathy was present on fundus examination [Figure 1]. The left eye, on examination, was within normal limits with the exception of anterior lenticonus, posterior subcapsular cataract, and fleck retinopathy. Direct ophthalmoscopy revealed an oil droplet reflex bilaterally [Figure 2]. The diagnosis of Alport syndrome was made and the patient was advised to seek additional care from a nephrologist and an ear, nose, and throat specialist. Eighteen months later, the patient developed progressive posterior lenticonus bilaterally [Figure 3]. Progression of the cataract in the left eye was also noted. The manifest refraction changed to –4.00 –1.00 × 85 and –8.00 –0.75 ×125 in the right and left eyes, respectively. One year after his initial presentation, the patient underwent renal transplant for his chronic renal failure. The patient was kept on regular follow-up visits as the decision for cataract surgery was delayed as the patient’s vision was not significantly affected.

## DISCUSSION

Some authors have considered anterior lenticonus as a manifestation of Alport syndrome, whereas posterior lenticonus is not associated with systemic disease.^5^ Others have suggested that posterior lenticonus is a rare manifestation of Alport syndrome.^2^–^4^ The prevalence of posterior lenticonus is estimated at 1–4 in 100,000 children.^6^

The ocular and clinical features of Alport syndrome are identical in both the X-linked and autosomal recessive forms. Retinopathy and cataracts are the only ocular abnormalities described in the rare autosomal dominant form of Alport syndrome.^1^ Previous literature has reported variability in the incidence of ocular manifestations of Alport syndrome. Colville *et al*.^1^ reported dot-and-fleck retinopathy in 85% of affected adult males, anterior lenticonus in approximately 25%, and with posterior polymorphous corneal dystrophy being rare. Chugh *et al*.^7^ reported the prevalence of anterior lenticonus in 37.8% of the subjects, retinal flecks in 22.2%, cataract in 20%, and keratoconus in 6.7% of subjects. Teekhasaenee *et al*.^8^ reported ocular manifestations in 82.3% of subjects with Alport syndrome. Posterior polymorphous dystrophy was the most common manifestation, occurring in 64.7% of subjects. Jacobs *et al*.^9^ reported that ocular changes are uncommon and subtle in young patients with Alport syndrome, and that the signs increase in frequency and severity with age. In our case, both anterior lenticonus and cataract were present. The presence of posterior lenticonus late in the natural history of the syndrome may reflect the progressive nature of this manifestation.

The X-linked mutations have been mapped to defects in the α-5-chain of the type IV collagen gene, compromising the COL4A5, COL4A3, and COL4A4 genes. All mutations lead to abnormalities in the basement membrane of the glomerulus, cochlea, retina, lens capsule, and cornea, which eventually contribute to the typical phenotype of Alport syndrome.^1^ Recently, the presence of a complex (core plus secondary) binding site for TCF8 in the promoter of Alport syndrome gene COL4A3, which encodes collagen type IV alpha 3 is thought to cause posterior polymorphous dystrophy.^10^

As Alport syndrome represents a mutation coding for collagen type IV, the lens capsule is considerably thinner. Junk *et al*.^11^ conducted an electron microscopy study of a fragile capsulorhexis specimen and found typical thinned basal lamina with basement membrane disruptions. Similar findings were reported by Citirik *et al*.,^12^ Kato *et al*.,^13^ and Takei *et al*.^14^ Choi *et al*.^15^ reported the presence of numerous vascular dehiscences localized at the inner part of the lens capsule, large numbers of capsular dehiscences containing fibrillar materials, and vacuoles in addition to decreased thickness of the anterior lens capsules (4–13 *μ*m).

The presence of cataract, either as a component of the disease or as a side effect of oral steroids following renal transplant, together with a fragile capsule makes cataract surgery more challenging. Phacoemulsification has been reported as a safe procedure in such cases. Zare *et al*.^16^ reported good results with phacoemulsification and implantation of a single-piece acrylic hydrophobic intraocular lens (IOL) in 11 eyes of 6 patients with Alport syndrome. Seymenoğlu *et al*.^17^ and Aslanzadeh *et al*.^18^ reported the safety of phacoemulsification with IOL implantation in 8 and 4 eyes, respectively. Anterior capsular rupture either spontaneously^19^,^20^ or following trauma^21^ have been reported. In order to perform a continuous curvilinear capsularhexis (CCC) and avoid inadvertent spontaneous capsular rupture, several maneuvers have been reported. Van Setten *et al*.^22^ reported performing cortical lens matter aspiration prior to performing CCC. Zare *et al*.^16^ and Jaspreet *et al*.^3^ reported performing a CCC starting at the midperiphery rather than a conventional CCC, which is initiated at the center of the capsule. A pars plana approach for cataract surgery in the posterior lenticonus has also been reported in a single case with good outcome.^23^

In conclusion, Alport syndrome affects multiple systems, including the eye. The ocular manifestations are important to recognize in order to determine the proper medical and surgical therapy. Posterior lenticonus, which was once considered as an isolated manifestation is being reported more frequently in association with Alport syndrome, suggesting that posterior lenticonus is part of the disease.
